# Stability Evaluation of Reference Genes in *Gynaephora qinghaiensis* (Lepidoptera: Lymantriidae) for qRT-PCR Normalization

**DOI:** 10.3390/insects16101019

**Published:** 2025-10-01

**Authors:** Honggang Li, Fengmei Chang, Xiaoning Cui, Boxin Xi, Guangwei Li, Deguang Liu, Kuiju Niu

**Affiliations:** 1 Key Laboratory for Grassland Ecosystem of Education Ministry, College of Pratacultural, Gansu Agricultural University, Lanzhou 730070, China; lhg2000626@gmail.com (H.L.); cfm231620@gmail.com (F.C.); sxqxbx@outlook.com (B.X.); niukj@gsau.edu.cn (K.N.); 2 College of Life Science, Yan’an University, Yan’an 716000, China; liguangwei@yau.edu.cn; 3 College of Plant Protection, Northwest A&F University, Yangling 712100, China; dgliu@nwsuaf.edu.cn

**Keywords:** *Gynaephora qinghaiensis*, qRT-PCR, normalization, reference genes, gene expression

## Abstract

The grassland caterpillar *Gynaephora qinghaiensis* (Lepidoptera: Lymantriidae) is a dominant pest species in the alpine meadows of the Tibetan Plateau. Elucidating changes in key gene expression patterns will provide molecular insights into the adaptive evolutionary mechanisms of insects. Quantitative real-time PCR (qRT-PCR) is currently the predominant analytical methodology for assessing gene expression levels. However, variability among samples can compromise result reliability. Thus, selecting stably expressed reference genes for target gene normalization under diverse scenarios is critical. To date, suitable reference genes for *G. qinghaiensis* under varying experimental conditions have remained unidentified. In this study, the transcriptome data of *G. qinghaiensis* were obtained using the RNA-seq technique, and 13 candidate reference genes were selected. Four independent algorithms—ΔCt, geNorm, NormFinder, and BestKeeper—as well as a comprehensive online platform, RefFinder, were employed to evaluate the stability under six experimental conditions (tissues, developmental stages, sexes, temperatures, starvation, and insecticide treatments). Our findings identified the following optimal reference gene combinations for each experimental condition: *RPS18*, *RPS15*, and *RPL19* for tissue samples; *RPL19*, *RPS15*, and *RPL17* across developmental stages; *RPS18* and *RPS15* for different sexes; *RPS8* and *EF1-α* under varying temperature conditions; *RPL17* and *RPL15* during starvation; and *RPL19* and *RPL17* following insecticide treatments. To validate the feasibility of the reference genes, we examined the expression of the target gene *HSP60* in different tissues and under different temperatures. Our results established essential reference standards for qRT-PCR with *G. qinghaiensis* samples, laying the foundation for precise gene expression quantification in the future.

## Simple Summary

The selection of appropriate reference genes is critical for normalizing quantitative reverse transcription polymerase chain reaction data and directly enhances the accuracy of target gene quantification. However, the stable reference genes for *Gynaephora qinghaiensis* under diverse experimental conditions remain unknown. Therefore, we selected 13 candidate reference genes and evaluated their stability across different tissues, sexes, developmental stages, temperatures, starvation states, and insecticide treatments. The results were further validated by expression levels of the target gene *HSP60* under varying tissue and temperature conditions. The results demonstrate that the optimal combination of reference genes is as follows: *RPS18*, *RPS15*, and *RPL19* for tissues; *RPL19*, *RPS15*, and *RPL17* for developmental stages; *RPS18* and *RPS15* for sexes; *RPS8* and *EF1-a* for temperatures; *RPL17* and *RPL15* for starvation conditions; and *RPL19* and *RPL17* for insecticide treatments. Our results will lay a foundation for future studies on gene expression and function in *Gynaephora qinghaiensis*.

## 1. Introduction

*Gynaephora* species are endemic to the Tibetan Plateau, which can survive in extreme climatic conditions, including hypoxia, low temperatures, and intense ultraviolet radiation [1]. The grassland caterpillar *Gynaephora qinghaiensis* is one of the important species widely distributed in the Qinghai, Tibet, Gansu, and Sichuan provinces in China [1]. Its larvae primarily feed on Cyperaceae (e.g., *Kobresia humilis*, *Kobresia parva*) and Poaceae (e.g., *Elymus nutans*, *Festuca rubra*) and also cause damage to Asteraceae (e.g., *Saussurea pulchra*, *Leontopodium nanum*) and Rosaceae (e.g., *Potentilla anserina*, *Potentilla bifurca*) plants. Over 20 various plant species have been documented as its hosts [1]. Additionally, *Gynaephora* larvae and cocoons are poisonous, commonly causing oral mucosal lesions and broken tongue diseases in livestock animals [2,3]. So, it can greatly reduce the quality and yield of edible forage grass in alpine meadows, thereby influencing animal husbandry. At present, bioinsecticides, including fungi, bacteria, viruses, and plant-derived insecticides, are used for *Gynaephora caterpillar* control. Increasing molecular research has been conducted on *Gynaephora*, such as species classification and adaptive evolutionary mechanisms of populations [4,5,6,7]. Chemical perception in courtship and mating behaviors of its male and female adults has also been reported [8,9]. The advancements of molecular biology of *Gynaephora* may promote our understanding of the occurrence patterns and resistance formation mechanisms, facilitating the development of green management strategies by integrating the use of key functional genes.

Although RNA sequencing (RNA-seq) is increasingly popular, quantitative reverse transcription polymerase chain reaction (qRT-PCR) has remained a common technique due to its accuracy and high sensitivity to determine small-scale gene expression levels [10]. However, several factors may compromise the accuracy and reliability of qRT-PCR results, including RNA sample integrity, amplification efficiency, pipetting errors, variation among different biological samples, and so forth [11,12,13]. Therefore, it is necessary to utilize suitable reference genes under specific environmental stresses to normalize target gene expression data using the 2^−ΔΔCt^ method [14].

Previous studies have demonstrated that candidate reference genes usually exhibit variable expression stability across different experimental conditions. There is not a single reference gene identified that can maintain consistent expression across all environmental conditions, even within the same insect species [13,15,16,17,18]. For example, screening suitable reference genes in *Spodoptera litura* is necessary under diverse conditions, encompassing both biotic factors (developmental stage, tissue, and population) and abiotic treatments (temperature, insecticide, diet, and starvation) [19,20,21]. Furthermore, insufficient and excessive numbers of reference genes can also influence the reliability of data normalization [22]. With the expanding scope of molecular ecological research on *G. qinghaiensis*, selecting appropriate reference genes for diverse environmental conditions is essential to guarantee accurate quantification of target gene expression levels.

Although Zhang et al. identified *EF1-α*, *RPS15*, and *RPS13* as the most stable reference gene combination for *Gynaephora* under an altitudinal gradient [23], research on other experimental conditions remains limited. In this study, we evaluated the expression stability of 13 commonly used reference genes in *G. qinghaiensis*, including arginine kinase (*AK*), elongation factor 1 alpha (*EF1-α*), glyceraldehyde-3-phosphate dehydrogenase (*GAPDH*), *Troponin C*, alpha tubulin (*α-Tub*), *Cyclin A*, ribosomal protein L7 (*RPL7*), ribosomal protein L15 (*RPL15*), ribosomal protein L17 (*RPL17*), ribosomal protein L19 (*RPL19*), ribosomal protein S8 (*RPS8*), ribosomal protein S15 (*RPS15*) and ribosomal protein S18 (*RPS18*). The evaluations were conducted under six experimental conditions (adult tissues, developmental stages, sexes, temperature variations, starvation stresses, and insecticide exposures) using five statistical algorithms: the ΔCt method, BestKeeper, geNorm, NormFinder, and RefFinder. To further verify our results, the expression profile of the heat shock protein 60 (*HSP60*) gene was determined. The present study provides a basis for further understanding the molecular mechanisms of gene expression-mediated plasticity and insecticide resistance development involving *G. qinghaiensis*.

## 2. Materials and Methods

### 2.1. Insect Collection and Rearing

Larvae of *G. qinghaiensis* were collected in August 2024 from alpine meadows in Maqu County, Gansu Province, China (34.01° N, 102.04° E; elevation 3501 m asl). The larvae were fed with fresh leaves of *Elymus nutans* until adulthood in an incubator (HQH-H500, Shanghai Yuejin, Inc., Shanghai, China) maintained at 21 ± 1 °C, relative humidity of 65 ± 5%, and a photoperiod of 16 h: 8 h (light: dark). Newly emerged adults were provided with 5% honey as a daily dietary supplement. Three-day-old unmated adults were used in the experiment. The identification of male adults and larval instars was based on the method described by Yan et al. [24].

### 2.2. Sample Preparation

To evaluate the expression stability of reference genes under different conditions, six experimental groups were established: tissue, developmental stage, sex, temperature, starvation, and insecticide.

For tissue samples, 80 antennae, 20 heads, 20 thoraxes, 10 abdomens, 120 legs, and 40 wings were dissected and collected separately in male adults. For developmental stages, samples were collected as follows: 300 eggs, one fifth-instar larva, one pupa, and one male adult. For comparison between sexes, one adult individual was used in each sample. In studies of multiple stress conditions, fifth-instar larvae were employed. For the temperature treatment groups, larvae were exposed to five temperatures (11 °C, 16 °C, 21 °C, 26 °C, and 31 °C) for 9 h, with a healthy larva collected as a biological replicate. For the starvation treatment group, larvae were deprived of food for 24 h, and healthy larvae were collected. For the insecticide treatment groups, we selected insecticides with proven efficacy against Lepidoptera species, based on previous research. Larvae were exposed to fresh leaves treated with four insecticides through the leaf impregnation methodology [21]: *Beauveria bassiana* (*B. bassiana*) (0.6 × 108 spores·mL^−1^), rotenone (3% *v*/*v*), abamectin (2.5% *v*/*v*), and cypermethrin (1.5% *v*/*v*) [21,25,26]. Following 48 h of continuous feeding, the surviving larvae were collected.

Following collection, the samples were transferred to 1.5 mL centrifuge tubes and immediately snap-frozen in liquid nitrogen for RNA extraction. Each tube contained a single insect specimen, with three independent biological replicates being processed for each experimental group.

### 2.3. RNA Extraction and cDNA First Strand Synthesis

RNA was isolated from all samples using the TRIzol reagent (Ambion, Austin, TX, USA) according to the manufacturer’s protocol [27,28]. The concentrations and OD_260/280_ values of RNA samples were measured using a Nanodrop 2000 spectrophotometer (Thermo Scientific, Waltham, MA, USA), and the integrity was analyzed with electrophoresis on 1.5% agarose gels. [29,30] RNA samples demonstrating optimal OD_260/280_ ratios (1.9–2.1) and exhibiting clear and single electrophoretic bands on 1.5% agarose gels were selected for subsequent analyses. First-strand cDNA synthesis was performed using 500 ng of qualified RNA with the SweScript All-in-One RT SuperMix kit (Servicebio, Wuhan, China) following the manufacturer’s protocol. The synthesized cDNA products were aliquoted and stored at −20 °C until further use.

### 2.4. RNA Sequencing and Selection of Candidate Reference Genes

Transcriptome sequencing of *G. qinghaiensis* was performed on four groups, namely fifth-instar larvae, male adults, female adults, and male adult antennae, each containing three biological replicates. The total RNA was isolated using the TRIzol reagent, followed by enzymatic elimination of genomic DNA contamination with RNase-free DNase I (Takara, Kusatsu, Japan). RNA preparations were shipped to Beijing Allwegene Technology Co., Ltd. (Beijing, China) for the RNA-Seq library construction and sequencing (NovaSeq 6000 platform, PE150) (Illumina, San Diego, CA, USA). The Trinity v. 2.4.0 software was used for transcript assembly. A total of 51,124 unigenes were assembled from the RNA-seq data.

The candidate reference genes were selected from previously reported research [13]. The gene sequences were derived from the *G. qinghaiensis* transcriptome database (bioproject accession number: PRJNA1307161), and complete open reading frames (ORFs) were confirmed using the NCBI ORFfinder function (https://www.ncbi.nlm.nih.gov/orffinder/, accessed on 25 November 2024). Subsequently, candidate genes were retrieved through BLAST homology analysis against the transcriptomic database, and the detailed information can be found in Table 1. A total of 13 candidate reference genes (*AK*, *EF1-α*, *GAPDH*, *RPL7*, *RPL19*, *RPS15*, *RPS18*, *Troponin C*, *α-Tub*, *Cyclin A*, *RPL15*, *RPL17*, and *RPS8*) were selected for qRT-PCR.

### 2.5. Primer Design and qRT—PCR Analysis

Specific primers for 13 candidate reference genes were designed using Primer Premier 5.0 (Table 2). Initially, we performed qRT-PCR using male adult cDNA as the template to test the amplification efficiency and specificity of each primer pair. The qRT-PCR reactions were set up using 2× SYBR Green qPCR Master Mix (Servicebio, Wuhan, China) on a LightCycler^®^ (Roche, Basel, Switzerland). The total reaction system was 20 μL, including 10 μL 2× Universal Blue SYBR Green qPCR Master Mix, 0.4 μL of upstream and downstream primers, 1 μL of cDNA template, and 8.2 μL of RNase-free sterile water. All reactions were conducted with three biological replicates and two technical replicates using 96-well optical PCR plates, with subsequent sealing through optical sealing tape. The reaction protocols are as follows: one cycle at 95 °C for 30 s, followed by 40 cycles: denaturation at 95 °C for 15 s, annealing at 60 °C for 30 s (a two-step method). The melt curve analysis was as follows: the temperature ranged from 55 °C to 95 °C, with an increase of 0.5 °C per cycle. The SYBR green signal intensities were simultaneously measured. Real-time monitoring of SYBR Green fluorescence intensity was implemented synchronously with other parameters. To calculate the amplification efficiency (*E*), correlation coefficient (*R*^2^), and linear regression equation, each primer pair was run on five-fold serial dilutions of cDNA templates with concentrations of 200 ng/μL, 40 ng/μL, 8 ng/μL, 1.6 ng/μL, and 0.32 ng/μL. E value was calculated using the following formula: E = [10^(−1/slope)^ − 1] × 100%. *R*^2^ was the slope of the amplification curve.

Following validation of primer specificity, the expression profiles of 13 candidate reference genes under six experimental conditions were obtained by qRT-PCR analyses using all cDNA samples (at a concentration of 150 ng/μL). The reaction conditions remained the same as the previously established reaction parameters.

### 2.6. Statistical Analyses

Four independent algorithms (ΔCt, geNorm, NormFinder, and BestKeeper) and the web-based RefFinder platform (https://blooge.cn/RefFinder/, accessed on 4 March 2025) [31,32,33] were employed to assess and rank candidate reference genes for identifying the most stable internal controls under various experimental conditions.

The ΔCt method evaluates gene expression stability by analyzing cycle thresholds (Ct), prioritizing genes with lower standard deviations (SD) in Ct values as more stable reference candidates. GeNorm and NormFinder algorithms both assess gene stability using 2^−ΔCt^ data (ΔCt = each Ct-minimum Ct) to generate stability values (M), where a lower M value indicates higher stability. GeNorm can calculate pairwise variation (V) to determine optimal reference gene numbers, but NormFinder employs distinct computational methods and cannot identify the number of ideal reference genes. BestKeeper is a computational tool that evaluates reference gene stability directly from Ct values using parameters like SD and CV, with lower values indicating greater stability. RefFinder is an online platform that employs four algorithms (ΔCt, geNorm, NormFinder, and BestKeeper) to assess reference gene suitability, generating unified stability rankings by combining algorithm-specific results with weighted adjustments based on gene reliability.

### 2.7. Validation of Reference Genes with the Target Gene HSP60

To assess the expression stability of candidate reference genes, *HSP60* (upstream primer: 5′-GGAAATGGCAAACCAGCAG-3′ and downstream primer: 5′-GATTACACCGCCCGTAGCA-3′) was selected for the purpose of verification. The expression patterns of *HSP60* were quantitatively analyzed using qRT-PCR in both adult male tissues and temperature-stressed larvae. Ct values were subsequently normalized through the 2^−ΔΔCT^ method [34] using three different sets of reference genes: the most stable reference genes, the recommended reference combination, and the least stable ones. One-way analysis of variance (ANOVA) was used to compare the gene expression levels of *HSP60*, followed by Tukey’s multiple comparison analyses in SPSS v. 28.0 (IBM Corp., Armonk, NY, USA).

## 3. Results

### 3.1. Specificity of Primers of Candidate Reference Genes

**Figure 1:**
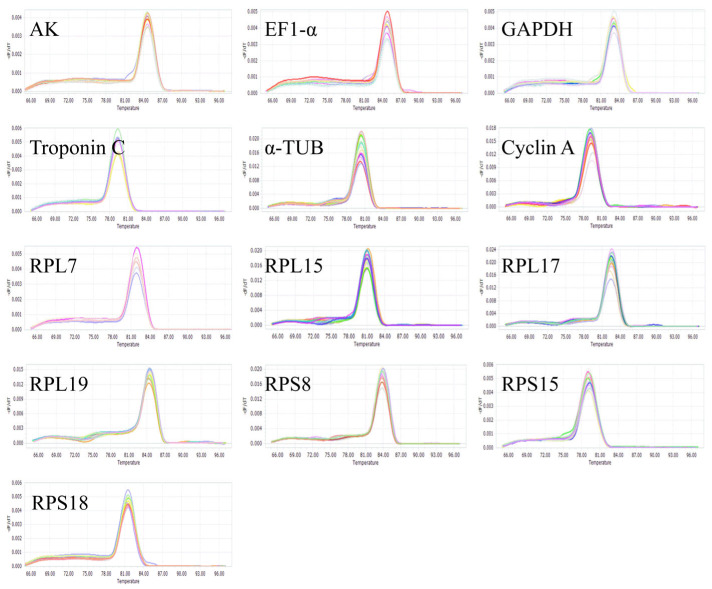
Melting curves of 13 candidate reference genes for RT-qPCR amplification of *Gynaephora qinghaiensis* samples.

The specificity of 13 candidate reference gene primers was tested by qRT-PCR. The melting curves of each gene primer pair exhibited a single peak (Figure 1). The standard curves of all candidate reference genes were constructed based on different concentration gradients, revealing amplification efficiency ranging from 90.2% to 105.2%, and correlation coefficient (*R*^2^) between 0.97 and 0.99 (Table 2). These findings indicated that the primers possessed high specificity.

### 3.2. Expression Profiles of Candidate Reference Genes

**Figure 2:**
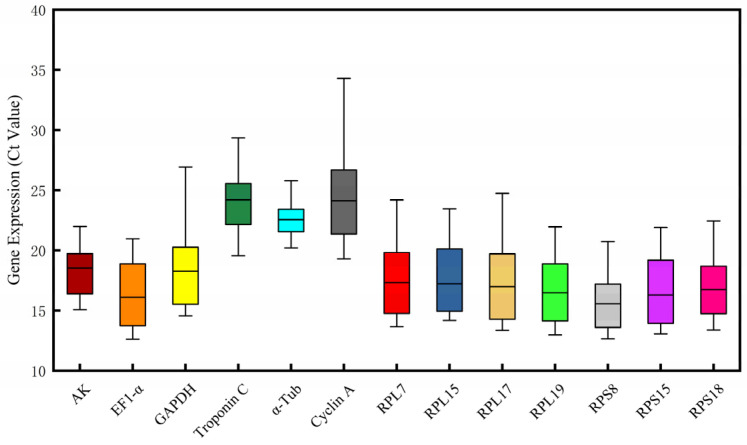
Expression profiles of 13 candidate reference genes for all samples of *Gynaephora qinghaiensis* (mean cycle threshold (Ct) values obtained under different experimental conditions).

The expression profiles of 13 candidate reference genes were analyzed to assess the overall variability among different reference genes. The results demonstrated a broad expression range among the 13 candidate reference genes, with Ct values spanning from 12.61 (*EF1-a*) to 34.3 (*Cyclin A*). Notably, *Troponin C* and *Cyclin A* were identified as genes with the lowest expression levels, whereas *RPS8* exhibited the highest expression. Among all test genes, α-Tubulin (*α-Tub*) showed the least variability, whereas *Cyclin A* displayed the greatest variation (Figure 2). These findings underscore the notion that no single universal reference gene can be universally applicable across all experimental conditions.

### 3.3. Expression Stability of Candidate Reference Genes Under Six Different Experimental Conditions

#### 3.3.1. Tissues

Using the ΔCt, geNorm, and BestKeeper methods, the top three ranked reference genes were identified as *EF1-α*, *RPL19*, and *RPS15* (Figures S1A and S2A; Table 3). Normfinder showed the most stable reference gene as *RPS18*, consistent with the results of RefFinder (Figure S3A; Table 4). Notably, all analytical methods indicated that *AK* was the least stable reference gene under different tissue treatments (Table 4).

#### 3.3.2. Developmental Stages

Based on the ΔCt and BestKeeper analyses, α-Tub was identified as the most stable reference gene (Figure S1B; Table 3). However, the results from geNorm and Normfinder analyses differed, with both methods indicating that *RPS15*, *RPL17*, and *RPL19* were the top three reference genes across different developmental stages (Figures S2B and S3B). The analyses with RefFinder produced similar results (Table 4).

#### 3.3.3. Sexes

For sexes, *GAPDH* was identified as the most stable reference gene by both the ΔCt and BestKeeper (Figure S1C; Table 3). With Normfinder, *RPS18* was the most stable reference gene (Figure S3C), whereas *RPS15* was the most stable one with geNorm (Figure S2C). A comprehensive analysis using RefFinder concluded that the top three reference genes were *RPS18*, *RPS15*, and *RPL19*. Notably, all methods consistently identified *Cyclin A* as the least stable reference gene (Table 4).

#### 3.3.4. Temperatures

The comprehensive evaluation revealed the stability ranking of reference genes under different temperatures as follows: *RPS8* > *EF1-α* > *RPS18* > *RPL15* > *RPL19* > *RPL7* > *α-Tub* > *RPL17* > *GAPDH* > *AK* > *RPS15* > *Cyclin A* > *Troponin C* (Table 4). Both the ΔCt method and BestKeeper identified *EF1-α* as the top-ranked reference gene (Figure S1D; Table 3), while geNorm ranked *RPS18* as the most stable (Figure S2D), and Normfinder designated *RPS8* as the most stable (Figure S3D). Notably, *Troponin C* was consistently identified as the least stable reference gene across all computational programs.

#### 3.3.5. Starvation Treatments

*RPL17* was identified as the most stable reference gene with RefFinder (Table 4). Both ΔCt and BestKeeper produced similar rankings for reference genes, with *α-Tub*, *RPS18*, and *RPL15* being the top three ranked reference genes (Figure S1E; Table 3). Conversely, geNorm revealed that *RPL15* and *RPS8* were the most stable ones (Figure S2E).

#### 3.3.6. Insecticides

Under four different insecticide treatments, the stability ranking of 13 reference genes with RefFinder was as follows: *RPL19* > *RPL17* > *RPS8* > *RPS18* > *RPL7* > *RPS15* > *GAPDH* > *EF1-α* > *RPL15* > *AK* > *α-Tub* > *Troponin C* > *Cyclin A* (Table 4). With the ΔCt method, *RPL17*, *RPL19*, and *RPS8* were identified as the top three most stable reference genes (Figure S1F), similar to the results with BestKeeper (Table 3). However, in Normfinder, *RPL19*, *RPS18*, and *RPL17* were ranked as the top three (Figure S3F). In contrast, geNorm analyses indicated that *RPS8*, *RPL7*, and *RPL17* were the top three most stable reference genes (Figure S2F).

### 3.4. Optimal Combinations of Reference Genes Under Six Experiment Conditions

**Figure 3:**
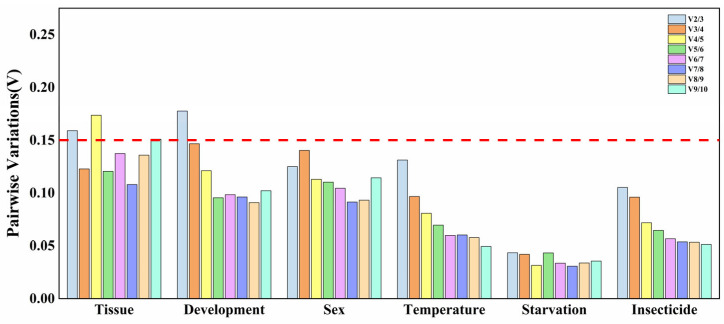
Optimal numbers of genes for normalization in *Gynaephora qinghaiensis* by gerNorm. The algorithm employs a threshold system (default V_n_/V_n+1_ < 0.15, adjustable per experimental requirements) to establish the minimally required reference genes: inclusion terminates (n) when V_n_/V_n+1_ falls below the threshold, whereas exceeding this value necessitates (n + 1) reference genes.

Utilizing multiple reference genes for normalization leads to a more precise analysis of gene expression levels. In geNorm, the pairwise variation (V_n_/V_n+1_) is used to determine the number of reference genes required, with a threshold value of 0.15. The results indicate that, in different tissues and developmental stages, V_2/3_ > 0.15 and V_3/4_ < 0.15, suggesting that three reference genes were suitable. Under other conditions, V_2/3_ values were lower than 0.15, indicating that two reference genes were sufficient (Figure 3). By integrating the rankings of stability provided via RefFinder for 13 reference genes under various experimental conditions, the optimal combinations of reference genes were identified for different experimental scenarios: *RPS18*, *RPS15*, and *RPL19* for different tissues; *RPL19*, *RPS15*, and *RPL17* for different developmental stages; *RPS18* and *RPS15* for different genders; *RPS8* and *EF1-α* for temperature stress; *RPL17* and *RPL15* for starvation stresses; and *RPL19* and *RPL17* for insecticide stresses (Table 5).

### 3.5. Validation of Candidate Reference Genes in Different Tissues and at Different Temperatures

**Figure 4:**
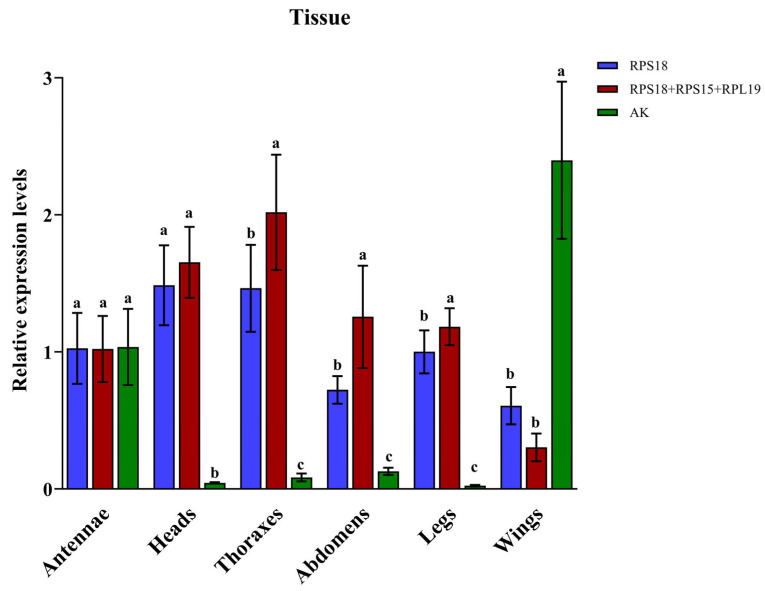
Validation of reference gene stability in different adult male tissues of *Gynaephora qinghaiensis*. Relative expression levels were normalized using *RPS18* (the most stable reference gene), *AK* (the least stable), and a combination of reference genes (*RPS18*, *RPS15*, and *RPL19*). Data in the figure are mean ± SE. Distinct letter labels indicate significant differences in *HSP60* expression levels among reference options within the same tissue (*p* < 0.05, one-way ANOVA).

**Figure 5:**
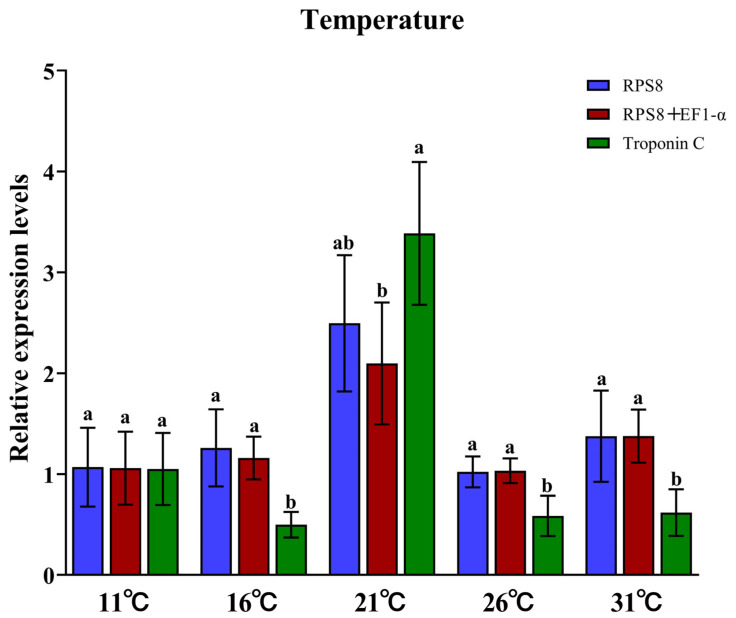
Validation of reference gene stability under different temperatures. Relative expression levels were normalized using *RPS8* (the most stable reference gene), *Troponin C* (the least stable), and a combination of reference genes (*RPS8*, *EF1-α*). Data in the figure are mean ± SE. Distinct letter labels indicate significant differences in *HSP60* expression levels within the same temperature when normalized using different reference genes (*p* < 0.05, one-way ANOVA).

Bar graphs (Figure 4 and Figure 5) clearly show that expression profiles normalized with the most stable reference gene and the recommended combination exhibited similar trends across both experimental treatments. In contrast, significant deviations in expression patterns were observed when normalization relied on the least stable reference gene.

The expression patterns of *HSP60* across different male adult tissues exhibited substantial variation depending on the reference genes selected for normalization. When employing *RPS18* (the most stable reference gene) or the combination of *RPS18*, *RPS15*, and *RPL19* as internal controls, the highest *HSP60* expression levels were detected in heads and thoraxes, with the lowest expression observed in wings. Conversely, normalization against *AK* (the least stable reference gene) led to maximal *HSP60* expression in wings and minimal expression in legs. Notably, compared to normalization with *AK*, *HSP60* expression patterns in heads, thoraxes, abdomens, legs, and wings showed significant differences (heads, F_2,15_ = 92.875, *p* < 0.001; thoraxes, F_2,15_ = 64.214, *p* < 0.001; abdomens, F_2,15_ = 37.993, *p* < 0.001; legs, F_2,15_ = 163.538, *p* < 0.001; wings, F_2,15_ = 64.459, *p* < 0.001) when normalized using either *RPS18* alone or the recommended gene combination.

When normalizing *HSP60* expression levels using the most stable single gene (*RPS8*), the recommended combination (*RPS8* + *EF1-α*), and the least stable gene (*Troponin C*), the highest expression was consistently detected at 21 °C. Normalization with *RPS8* alone and the *RPS8* + *EF1-α* combination revealed minimal expression at 26 °C, whereas the lowest expression was found at 16 °C when using *Troponin C*. The normalized expression levels of *HSP60* using three reference genes exhibited significant differences under 16 °C, 21 °C, 26 °C and 31 °C (16 °C, F_2,15_ = 14.92, *p* < 0.001; 21 °C, F_2,15_ = 5.935, *p* < 0.05; 26 °C, F_2,15_ = 14.892, *p* < 0.001; 31 °C, F_2,15_ = 10.513, *p* < 0.05). Notably, at 16 °C, 26 °C, and 31 °C, both *RPS8* and the combined *RPS8* + *EF1-α* reference genes led to significantly higher expression values compared with *Troponin C*. However, at 21 °C, only the combination of *RPS8* + *EF1-α* demonstrated significantly different expressions relative to *Troponin C*.

## 4. Discussion

Elucidating gene expression profiles under biotic and abiotic stress conditions will provide a molecular explanation for *Gynaephora* species’ specific adaptive strategies and evolutionary processes. Using appropriate reference genes can enhance the accuracy of target gene expression quantification, which is crucial for normalizing qRT-PCR data [35]. In this study, we selected and evaluated 13 commonly used reference genes in *G. qinghaiensis* under varying experimental conditions (male adult tissues, developmental stages, sexes, temperature treatments, starvation treatments, and insecticide exposures) to obtain suitable reference genes for target gene expression normalization.

Several computational tools based on distinct statistical principles, such as geNorm, NormFinder, BestKeeper, and the ΔCt method, are available to evaluate the stability of reference genes, but their application to the same dataset may produce differing gene stability rankings. geNorm and NormFinder focus on the inherent stability of gene expression, with the former supporting multiple-gene combinations, whereas the latter is limited to single genes. In contrast, BestKeeper and the ΔCt method place greater emphasis on the variability of gene expression levels. To address the limitation of the individual methods, the RefFinder platform integrates the four aforementioned tools by assigning weights and calculating the geometric mean to generate a comprehensive ranking. The advantage of using this combined approach lies in utilizing the complementarity of multiple algorithms, thereby reducing bias and enhancing the robustness of the evaluation [33]. In this study, four independent analytical methods revealed a similar ranking of reference gene stability, with a particularly high degree of concordance observed between ΔCt and BestKeeper analyses. An increasing number of studies on Lepidoptera suggest that the use of multiple reference genes can enhance normalization accuracy, whereas excessive numbers of references may compromise results in reliability [22,36]. Consequently, the optimal gene combinations identified by geNorm effectively minimize the variability in qRT-PCR data, while RefFinder determines the most accurate stability rankings, thereby ensuring precise normalization. In this study, three reference genes were recommended for tissue and developmental stage analyses, whereas two genes were suggested for other experimental conditions. Our results balance methodological rigor with practical feasibility in data normalization.

In our study, based on six different experimental conditions for *G. qinghaiensis*, the most stable reference genes were all from the Ribosomal Protein (RP) family. RP genes are a class of genes that play a crucial role in cell metabolism and growth. Their metabolic products are evolutionarily conserved, which have been widely validated as reliable reference genes in many insect species [13,37,38,39,40,41,42]. Previous studies on *Bradysia odoriphaga* [43] and *Chlorops oryzae* [44] have identified *RPS15* as a stable reference gene during different developmental stages. Research on *Aphis glycines* [39] and *Plagiodera versicolora* [45] has validated *RPS18* for reliable normalization in different tissues. In this study, through the evaluation of multiple *RPS* and *RPL* subfamily genes, we found that the recommended reference genes were consistently derived from the RP family for all experimental treatments except under different temperature conditions. Notably, *RPS15* consistently ranked second in stability for different biological samples (tissues, developmental stages, and sexes), suggesting the potential for wide uses for RP family genes as a reference in *G. qinghaiensis*. The findings align with extensive evidence highlighting the robustness of *RPS* or *RPL* genes as normalization standards [32,43].

*EF1-α* is recognized as a crucial translational factor that catalyzes GTP-dependent binding of aminoacyl-tRNA to the ribosome’s acceptor site during protein synthesis [46]. Extensive previous research has shown that *EF1-α* can be widely used as a reference gene under multiple experimental conditions [39,47,48,49,50]. We identified *EF1-α* as a stable reference gene for *G. qinghaiensis* under varying temperatures. This finding parallels observations in other Lepidoptera species, such as *Spodoptera frugiperda* [51], *Spodoptera litura* [19], and *Danaus plexippus* [52]. *EF1-α* was similarly identified as an optimal reference gene for heat stress studies in *Chilo suppressalis* [53] and *Drosophila melanogaster* [46]. In this study, *EF1-α* was only applicable to different temperature treatments, reinforcing the notion that no universal reference gene was appropriate for all insect species or experimental conditions.

Chemical insecticides (e.g., pyrethroids) are widely implemented for *G. qinghaiensis* control. However, its repeated application has accelerated the development of pest resistance. Therefore, the rotational use of bioinsecticides can be a green and effective measure for their sustainable control. Investigation of target gene expression patterns under insecticide stress conditions could provide valuable insights for developing novel pest control strategies [54,55,56,57]. In this study, we obtained stable reference gene combinations under specific insecticide treatment conditions for the purpose of providing a basis for the analysis of related gene expression. Previous studies reported that *RPL32* and *RPL19* were stable reference genes in *Bactericera gobica* and *Phaedon brassicae* under chemical and bioinsecticide pressures, respectively [57,58]. These findings indicate that RPL family genes serve as stably expressed reference genes under insecticide stress conditions, which aligns with our results (*RPL19* and *RPL17*). The findings not only contribute to a deeper understanding of the defensive response mechanism and resistance evolution in grassland caterpillars under insecticide exposure but also offer support for the ecological assessment of chemical control impacts on the survival of non-target organism species in the Qinghai–Tibet Plateau.

The significance of molecular chaperones (heat shock proteins) in the regulation of normal cellular processes and thermotolerance has been extensively investigated [59]. To validate the effectiveness of recommended reference genes for use under treatments of different adult tissues and varying temperatures, we analyzed the expression profiles of *HSP60* with normalizations against the least stable reference gene, the most stable single reference gene, and optimal gene combinations. The relative transcript levels of *HSP60* displayed significant differences among the above-mentioned experimental conditions, similar to validation studies in other insect species [53,60]. For example, *HSP70* and *HSP60* gene expression levels were validated in *Aphis glycines*, *Plagiodera versicolora*, and *Chilo suppressalis* [39,45,53]. These findings underscore the pivotal role of appropriate reference gene selection for ensuring qRT-PCR data accuracy. Our findings also provide a solid foundation for functional studies of target genes in *G. qinghaiensis* under various environmental conditions.

## 5. Conclusions

We screened 13 commonly used reference genes for Lepidopteran insects from the transcriptome of *Gynaephora qinghaiensis*. The stability of these 13 reference genes was evaluated under six experimental conditions using ΔCt, geNorm, NormFinder, BestKeeper, and the comprehensive online platform RefFinder. The results demonstrated that distinct sets of reference genes were optimal for different experimental conditions: *RPS18*, *RPS15*, and *RPL19* for tissue comparisons; *RPL19*, *RPS15*, and *RPL17* for different developmental stages; *RPS18* and *RPS15* for comparing sexes; *RPS8* and *EF1-α* for temperature treatments; *RPL17* and *RPL15* for starvation conditions; and *RPL19* and *RPL17* for insecticide treatments. The reliability of the results was further validated by analyzing the expression patterns of the target gene *HSP60* across different tissues and under varying temperature conditions. Our results provide a basis for future research on gene expression and functional characterization in the *Gynaephora qinghaiensis*.

## Figures and Tables

**Table 1:** Sequence information and blast alignment data of candidate reference and target genes.

GenBank	Abbr	ORF (aa)	Blast Annotation	Acc. Number	E-Value	Identity (%)
PX137571	*AK*	226	arginine kinase [*Spodoptera litura*]	ADW94627.1	3 × 10^−154^	97.79%
PX137572	*EF1-α*	309	elongation factor 1-alpha [*Plodia interpunctella*]	XP_053609932.1	0	98.63%
PX137573	*GAPDH*	332	glyceraldehyde-3-phosphate dehydrogenase [*Lymantria dispar*]	QPZ44475.1	0	98.19%
PX137578	*Troponin C*	159	troponin C [*Helicoverpa armigera*]	XP_021199017.1	3 × 10^−91^	84.42%
PX137579	*α-Tub*	450	alpha-tubulin [*Bombyx mori*]	NP_001036885.1	0	99.78%
PX137580	*Cyclin A*	491	cyclin A [*Spodoptera frugiperda*]	AMY96431.1	0	81.61%
PX137574	*RPL7*	262	ribosomal protein L7 [*Spodoptera frugiperda*]	AAL62469.1	4 × 10^−163^	97.93%
PX137581	*RPL15*	204	ribosomal protein L15 [*Heliconius melpomene cythera*]	AEL28893.1	1 × 10^−118^	97.55%
PX137582	*RPL17*	187	ribosomal protein L17 [*Heliconius melpomene cythera*]	AEL28825.1	1 × 10^−109^	98.05%
PX137575	*RPL19*	200	ribosomal protein L19 [*Bombyx mori*]	NP_001037221.1	8 × 10^−106^	99.00%
PX137583	*RPS8*	198	ribosomal protein S8 [*Bombyx mori*]	NP_001037263.1	3 × 10^−112^	91.91%
PX137576	*RPS15*	147	ribosomal protein S15 [*Hyphantria cunea*]	WBO26470.1	2 × 10^−81^	99.32%
PX137577	*RPS18*	82	ribosomal protein S18 [*Bombyx mori*]	NP_001037269.1	2 × 10^−36^	100.00%
PX137584	*HSP60*	572	heat shock protein 60A [*Achroia grisella*]	XP_059057858.1	0	96.05%

**Table 2:** Primers and amplicon characteristics for candidate reference genes.

Gene Name	Primer Name	Primer Sequence (5′→3′)	Amplification SIZE (bp)	*E* (%)	*R* ^2^	Linear Regression Equation
*AK*	Forward Primer	CTTGGTACTACAGTCCGTGCG	150	91.0	0.98	Y = −3.5586X + 26
	Reverse Primer	GTCATAGACACCGCCTTCAGC				
*EF1-α*	Forward Primer	TGGAGCCCTCTACCAAAATG	127	90.2	0.98	Y = −3.5817X + 22.77
	Reverse Primer	TTGTCTGTGGGACGAGCAG				
*GAPDH*	Forward Primer	AACATAATCCCCGCCTCCAC	131	90.7	0.99	Y = −3.5663X + 24.97
	Reverse Primer	CGGACTGTGAGATCGACGAC				
*Troponin C*	Forward Primer	CATTCCCACATCAAGCCTCC	123	105.2	0.99	Y = −3.2036X + 30.25
	Reverse Primer	TCATCGAAATCCACGGTTCC				
*α-Tub*	Forward Primer	CAGGCTTATTGGACAGATCGTG	121	99.6	0.98	Y = −3.3326X + 29.72
	Reverse Primer	GGGGAAATGTATGCGAGGAT				
*Cyclin A*	Forward Primer	GGCACAGCCGCTACTTACA	141	94.6	0.97	Y = −3.4576X + 29.34
	Reverse Primer	GGACAGCACCTTCAGAATCAA				
*RPL7*	Forward Primer	CCTCCAGATCACCCTCAAGAG	191	91.2	0.99	Y = −3.5533X + 23.85
	Reverse Primer	GGATACGGATGACAAATGCG				
*RPL15*	Forward Primer	GTGTTGGTCGTCGTTGTGG	157	92.0	0.98	Y = −3.5311X + 23.05
	Reverse Primer	TTGTGAACTGCGTTGACTATCC				
*RPL17*	Forward Primer	AGACAGCAATGGCAATCAGG	192	90.6	0.98	Y = −3.5693X + 22.82
	Reverse Primer	GCAACTGGAGCAAGAACTCAG				
*RPL19*	Forward Primer	AGGAAGAAGGCTGAGAAGGC	107	91.2	0.99	Y = −3.5534X + 22.62
	Reverse Primer	CGCGAATGTCTGCAACAG				
*RPS8*	Forward Primer	TGGCATAAACGAAGGGCTAC	187	94.0	0.98	Y = −3.4751X + 22.96
	Reverse Primer	CAGATCCCCACGAGAAATTG				
*RPS15*	Forward Primer	GCTTTAGTGAAGAAACTCCGTCG	123	91.5	0.98	Y = −3.5454X + 22.71
	Reverse Primer	ACCAACAATAGAGCCAACCATC				
*RPS18*	Forward Primer	GTACAGCCAGCTAACCTCATCC	127	93.8	0.99	Y = −3.4804X + 23.41
	Reverse Primer	AGTATGTTGACCACGAACTCGG				

Note: “*E*” indicates the primer amplification efficiency; “*R*^2^” indicates the regression coefficient of the standard curve.

**Table 3:** Ranking of candidate reference genes for *Gynaephora qinghaiensis* using BestKeeper.

		*AK*	*EF1-α*	*GAPDH*	*Troponin C*	*α-Tub*	*Cyclin A*	*RPL7*	*RPL15*	*RPL17*	*RPL19*	*RPS8*	*RPS15*	*RPS18*
Tissure	SD	3.79	0.86	2.43	2.13	1.71	2.53	1.54	1.42	1.7	0.91	1.17	0.89	1.52
	CV	16.99	4.38	11.11	8.32	7.04	8.9	7.24	6.84	8.15	4.53	6.25	4.44	7.77
Development	SD	0.97	1.56	1.51	1.37	0.86	2.85	1.38	1.82	1.61	1.37	1.11	1.54	1.43
	CV	5.51	9.82	8.34	5.52	3.97	12.29	8.24	10.84	9.62	8.75	7.43	9.59	8.58
Sex	SD	0.66	0.72	0.32	1.52	0.42	2.39	0.84	1.43	0.84	0.43	0.32	0.75	0.53
	CV	3.22	3.98	1.57	5.65	1.81	9.16	4.41	7.59	4.43	2.38	1.93	4.14	2.81
Temperature	SD	0.51	0.25	0.39	0.79	0.31	0.51	0.38	0.34	0.57	0.45	0.37	0.4	0.43
	CV	3.1	1.88	2.52	3.31	1.41	2.34	2.5	2.28	3.86	3.01	2.66	2.84	2.8
Starvation	SD	0.3	0.43	0.62	0.63	0.06	0.28	0.48	0.17	0.19	0.27	0.22	0.36	0.14
	CV	1.86	3.04	3.81	2.9	0.27	1.35	3.24	1.16	1.37	1.98	1.58	2.66	1.02
Insecticide	SD	0.57	0.38	0.41	0.8	0.45	0.5	0.3	0.47	0.24	0.27	0.3	0.44	0.3
	CV	3.53	2.75	2.6	3.76	2.1	2.37	2.03	3.18	1.71	2	2.22	3.24	2.11

**Table 4:** Expression stability of candidate reference genes in *Gynaephora qinghaiensis* under different conditions.

Conditions	Gene	ΔCt	Rank	geNorm	Rank	Normfinder	Rank	BestKeeper	Rank	RefFinder
		SD		M		M		SD		Rank
Tissue	*AK*	4.235	13	1.587	13	1.869	13	3.79	13	13
	*EF1-α*	0.984	1	0.460	3	0.784	9	0.86	1	5
	*GAPDH*	2.806	11	1.096	10	0.682	5	2.43	11	9
	*Troponin C*	2.641	10	0.963	9	0.746	6	2.13	10	10
	*α-Tub*	2.305	9	1.362	12	1.168	12	1.71	9	11
	*Cyclin A*	2.997	12	1.236	11	0.963	11	2.53	12	12
	*RPL7*	2.032	7	0.860	8	0.788	10	1.54	7	8
	*RPL15*	1.830	6	0.723	6	0.334	2	1.42	5	4
	*RPL17*	2.187	8	0.808	7	0.414	3	1.7	8	7
	*RPL19*	1.124	2	0.366	1	0.771	7	0.91	3	3
	*RPS8*	1.333	4	0.510	4	0.471	4	1.17	4	6
	*RPS15*	1.147	3	0.366	1	0.781	8	0.89	2	2
	*RPS18*	1.822	5	0.671	5	0.204	1	1.52	6	1
Development	*AK*	1.230	2	0.853	10	0.638	10	0.97	2	10
	*EF1-α*	1.960	10	0.570	4	0.387	6	1.56	10	5
	*GAPDH*	1.902	9	0.621	5	0.378	5	1.51	8	4
	*Troponin C*	1.764	5	1.070	12	1.337	12	1.37	4	11
	*α-Tub*	1.096	1	0.912	11	0.730	11	0.86	1	6
	*Cyclin A*	3.654	13	1.299	13	1.721	13	2.85	13	13
	*RPL7*	1.766	6	0.643	6	0.417	7	1.38	6	8
	*RPL15*	2.249	12	0.781	9	0.492	9	1.82	12	12
	*RPL17*	1.997	11	0.353	1	0.237	3	1.61	11	3
	*RPL19*	1.726	4	0.492	3	0.124	1	1.37	4	1
	*RPS8*	1.343	3	0.689	7	0.441	8	1.11	3	7
	*RPS15*	1.858	8	0.353	1	0.202	2	1.54	9	2
	*RPS18*	1.781	7	0.735	8	0.366	4	1.43	7	9
Sex	*AK*	0.867	7	0.848	10	0.830	10	0.66	6	10
	*EF1-α*	0.807	6	0.547	5	0.258	3	0.72	7	7
	*GAPDH*	0.402	1	0.757	9	0.488	9	0.32	1	8
	*Troponin C*	1.666	11	1.155	12	1.599	12	1.52	12	12
	*α-Tub*	0.524	3	0.670	7	0.332	5	0.42	3	6
	*Cyclin A*	2.678	13	1.362	13	1.684	13	2.39	13	13
	*RPL7*	1.102	9	0.374	3	0.389	8	0.84	9	9
	*RPL15*	1.705	12	0.947	11	0.898	11	1.43	11	11
	*RPL17*	1.138	10	0.310	1	0.365	6	0.84	9	4
	*RPL19*	0.653	4	0.614	6	0.210	2	0.43	4	3
	*RPS8*	0.430	2	0.705	8	0.388	7	0.32	1	5
	*RPS15*	1.055	8	0.310	1	0.309	4	0.75	8	2
	*RPS18*	0.670	5	0.486	4	0.176	1	0.53	5	1
Temperature	*AK*	0.672	11	0.502	8	0.341	9	0.51	10	10
	*EF1-α*	0.297	1	0.466	6	0.189	2	0.25	1	2
	*GAPDH*	0.483	6	0.525	9	0.344	10	0.39	6	9
	*Troponin C*	0.984	13	0.668	13	0.670	13	0.79	13	13
	*α-Tub*	0.402	2	0.553	11	0.318	8	0.31	2	7
	*Cyclin A*	0.659	10	0.597	12	0.497	12	0.51	10	12
	*RPL7*	0.462	4	0.479	7	0.241	5	0.38	5	6
	*RPL15*	0.417	3	0.402	3	0.230	3	0.34	3	4
	*RPL17*	0.674	12	0.425	4	0.278	7	0.57	12	8
	*RPL19*	0.553	8	0.347	1	0.256	6	0.45	9	5
	*RPS8*	0.475	5	0.448	5	0.175	1	0.37	4	1
	*RPS15*	0.504	7	0.539	10	0.377	11	0.4	7	11
	*RPS18*	0.577	9	0.347	1	0.231	4	0.43	8	3
Starvation	*AK*	0.443	8	0.216	7	0.034	1	0.3	8	3
	*EF1-α*	0.572	10	0.251	9	0.140	6	0.43	10	9
	*GAPDH*	0.901	13	0.355	12	0.400	12	0.62	12	13
	*Troponin C*	0.837	12	0.318	11	0.347	11	0.63	13	12
	*α-Tub*	0.088	1	0.162	5	0.201	9	0.06	1	5
	*Cyclin A*	0.383	7	0.424	13	0.550	13	0.28	7	11
	*RPL7*	0.663	11	0.275	10	0.210	10	0.48	11	10
	*RPL15*	0.225	3	0.095	1	0.080	4	0.17	3	2
	*RPL17*	0.285	5	0.121	3	0.048	2	0.19	4	1
	*RPL19*	0.349	6	0.197	6	0.162	7	0.27	6	8
	*RPS8*	0.282	4	0.095	1	0.080	5	0.22	5	4
	*RPS15*	0.487	9	0.230	8	0.052	3	0.36	9	7
	*RPS18*	0.206	2	0.148	4	0.184	8	0.14	2	6
Insecticide	*AK*	0.643	12	0.471	9	0.300	9	0.57	12	10
	*EF1-α*	0.552	8	0.447	8	0.384	10	0.38	6	8
	*GAPDH*	0.507	6	0.429	7	0.260	6	0.41	7	7
	*Troponin C*	0.970	13	0.584	12	0.563	12	0.8	13	12
	*α-Tub*	0.598	10	0.530	11	0.401	11	0.45	9	11
	*Cyclin A*	0.611	11	0.639	13	0.590	13	0.5	11	13
	*RPL7*	0.415	5	0.270	1	0.280	7	0.3	3	5
	*RPL15*	0.556	9	0.494	10	0.284	8	0.47	10	9
	*RPL17*	0.305	1	0.317	3	0.201	3	0.24	1	2
	*RPL19*	0.371	2	0.371	4	0.066	1	0.27	2	1
	*RPS8*	0.401	3	0.270	1	0.234	5	0.3	3	3
	*RPS15*	0.508	7	0.409	6	0.216	4	0.44	8	6
	*RPS18*	0.414	4	0.387	5	0.119	2	0.3	3	4

**Table 5:** Recommended reference gene combinations in *Gynaephora qinghaiensis* for the different conditions.

Conditions	Reference Gene	Conditions	Reference Gene
Tissure	*RPS18*, *RPS15*, *RPL19*	Temperature	*RPS8*, *EF1-α*
Development	*RPL19*, *RPS15*, *RPL17*	Starvation	*RPL17*, *RPL15*
Sex	*RPS18*, *RPS15*	Insecticide	*RPL19*, *RPL17*

## Data Availability

The data presented in the study are deposited in the Figshare repository, accession number https://figshare.com/s/74bc4e0de4827e2f9092 (accessed on 20 August 2025).

## Supplementary Materials

The following supporting information can be downloaded at: https://www.mdpi.com/article/10.3390/insects16101019/s1, Figure S1: The stability of candidate reference genes for qRT-PCR of *Gynaephora qinghaiensis* samples based on ΔCt values; Figure S2: The stability of candidate reference genes for qRT-PCR of *Gynaephora qinghaiensis* samples with gerNorm; Figure S3: The stability of candidate reference genes for qRT-PCR of *Gynaephora qinghaiensis* samples with NormFinder.
